# A Novel Deep Learning Method to Predict Lung Cancer Long-Term Survival With Biological Knowledge Incorporated Gene Expression Images and Clinical Data

**DOI:** 10.3389/fgene.2022.800853

**Published:** 2022-03-14

**Authors:** Shuo Wang, Hao Zhang, Zhen Liu, Yuanning Liu

**Affiliations:** ^1^ College of Computer Science and Technology, Jilin University, Changchun, China; ^2^ Key Laboratory of Symbolic Computation and Knowledge Engineering of Ministry of Education, Jilin University, Changchun, China; ^3^ Graduate School of Engineering, Nagasaki Institute of Applied Science, Nagasaki, Japan

**Keywords:** cancer precision medicine, cancer survival prediction, CNN, deep learning, multimodal, survival analysis, optimal threshold selection

## Abstract

Lung cancer is the leading cause of the cancer deaths. Therefore, predicting the survival status of lung cancer patients is of great value. However, the existing methods mainly depend on statistical machine learning (ML) algorithms. Moreover, they are not appropriate for high-dimensionality genomics data, and deep learning (DL), with strong high-dimensional data learning capability, can be used to predict lung cancer survival using genomics data. The Cancer Genome Atlas (TCGA) is a great database that contains many kinds of genomics data for 33 cancer types. With this enormous amount of data, researchers can analyze key factors related to cancer therapy. This paper proposes a novel method to predict lung cancer long-term survival using gene expression data from TCGA. Firstly, we select the most relevant genes to the target problem by the supervised feature selection method called mutual information selector. Secondly, we propose a method to convert gene expression data into two kinds of images with KEGG BRITE and KEGG Pathway data incorporated, so that we could make good use of the convolutional neural network (CNN) model to learn high-level features. Afterwards, we design a CNN-based DL model and added two kinds of clinical data to improve the performance, so that we finally got a multimodal DL model. The generalized experiments results indicated that our method performed much better than the ML models and unimodal DL models. Furthermore, we conduct survival analysis and observe that our model could better divide the samples into high-risk and low-risk groups.

## 1 Introduction

As lung cancer is still a major contributor to cancer deaths, predicting lung cancer survival plays an important role in lung cancer precision medicine. Precision medicine is a novel kind of therapy which sprang up in the development of high-throughput sequencing technology and computer-aided treatment. It is able to give diseases a more detailed description by genomics and other technologies so that clinicians can get more precise targeted subgroups for therapies (Ashley, 2016), and survival prediction is one of the key components in precision medicine. Recent years have witnessed the burgeoning of sequencing data generation in the context of next-generation sequencing technology. RNA-Seq (Wang et al., 2009) was developed for profiling the transcriptome using deep-sequencing technologies, which can describe the transcripts far more precisely. A large amount of gene expression data was generated since its development.

As a result of the explosively increasing gene expression data, cancer analysis and prediction using gene expression data such as cancer survival prediction and cancer subtype prediction have become hot spots in biomedical research. Many machine-learning-based analysis methods had been proposed, such as survival trees (Gordon and Olshen, 1985), Bayesian methods (Fard et al., 2016), and artificial neural networks (ANNs) (Faraggi and Simon, 1995), so that pathological cancer analysis can be done at a molecular level and in a big-data background. With the fact that patients having the same disease still may give different responses to a specific therapy (Sharma and Rani, 2021), analyzing and dividing patients with the same disease according to their molecular-level features have the potential to improve diagnosis accuracy. In this paper, what we do can also be seen as to divide samples into different groups by the predicted survival status according to their gene expression data. There are many classical machine learning (ML) methods that have been widely used to make cancer prediction and analysis. For example, the Cox proportional hazard model is an algorithm which models the relationship between survival distribution and covariates with a proportional hazard assumption in a linear-like manner (Fox and Weisberg, 2002). Support vector machine (SVM) is a supervised ML algorithm that can be nicely summed up as (1) the separating hyperplane, (2) the maximum margin, (3) the soft margin, and (4) the kernel function (Noble, 2006). SVM has been used extensively by bioinformatics practitioners due to its powerful classification capability, such as gene selection for cancer classification (Guyon et al., 2002) and cancer survival prediction (Jiang et al., 2018). Besides the regression problem such as survival regression analysis and the classification problem such as cancer classification we have noted above, the unsupervised learning problems for complex objects with heterogeneous features are also ubiquitous and important in real-world applications (Ma and Zhang, 2019). For instance, some researchers leveraged the clustering method, an unsupervised ML algorithm, to predict survival and surgical outcomes with gene expression data and got reliable results (Wang et al., 2017).

Although ML algorithms are endowed with a natural ability to learn patterns automatically from data, they have some shortcomings. One of the greatest Achilles’ heels of classic ML methods is the strong dependence on how the data are represented. The classification performance of a machine model is closely related with the quality and relevance of the features. And deep learning (DL), as a part of the ML family, emerged to address this issue through automatically learning feature representations in the training process, thereby forming an end-to-end learning pipeline (Eraslan et al., 2019). And the unique compatibility with GPUs greatly facilitates the development of DL because of GPUs’ much higher computing performance than CPUs at similar prices. For the past few years, many bioinformaticians get into the combination between bioinformatics and DL. For instance, DeepBind was proposed in 2015, which leveraged the convolutional neural network (CNN) to predict the sequence specificities of DNA- and RNA-binding proteins using sequencing data. The results showed that it outperformed other state-of-the-art methods (Alipanahi et al., 2015). From that time, the usages of DL methods in bioinformatics have increased rapidly. Many novel DL models are applied in bioinformatics research and got great performance, such as the CNN we have noted above, LSTM (Lamurias et al., 2019), deep autoencoder (Chicco et al., 2014), and knowledge graph (Sousa et al., 2020).

Survival prediction is to build an association between covariates and the time of an event, and the covariates could be clinical information (for example, sex, cancer types, tumor stages, and ages), genomics data, and medical images; the time of event could be the time to death (overall survival, OS), the progression-free survival time (PFS), the disease-free survival (DFS), and the disease-specific survival (DSS). The canonical survival prediction methods are mainly some statistical ML algorithms such as Cox proportional hazard regression we have noted above, Kaplan–Meier estimator (Bland and Altman, 1998), and random survival forests (Ishwaran et al., 2008). Survival prediction plays an important role in bioinformatics research, and some researchers try to leverage the strong learning ability of DL for predicting survival patterns, such as DeepSurv (Katzman et al., 2018) and Cox-nnet (Ching et al., 2018). While DL methods have been widely used in recent years, they sometimes have difficulty in cancer survival prediction with genomics data due to the curse of dimensionality (Altman and Krzywinski, 2018), which means that, in cancer survival analysis and prediction problems, we usually have a small number of samples, namely, the patients; however, each sample has fairly high-dimensional features (for example, genes). Furthermore, the gene expression data are heterogeneous and noisy; many genes may be irrelevant with the target problem. All of the above factors usually cause the DL algorithms to become disoriented and more inclined to overfitting.

To address this “High Dimensionality, Few Samples” issue in cancer survival prediction, we design a DL method for cancer survival prediction. Firstly, we propose a method to convert patients’ gene expression data into two kinds of gene expression images, the first kind with KEGG BRITE (Kanehisa and Goto, 2000) gene functional information incorporated and the second kind with KEGG Pathway information incorporated, to overcome the curse of dimensionality. Then we propose a multimodal DL model with the two kinds of gene expression images and clinical data as inputs, to perform lung cancer long-term (60 months OS) survival prediction. Experiments on lung cancer data showed that our method achieved much better results on AUC (average AUC up to 71.48% on TCGA (Chang et al., 2013) lung cancer data set and 72.51% on GEO (Barrett et al., 2012) data set GSE37745 from 50 times experiments) than those of unimodal DL models and ML models. And survival analysis was conducted to further prove the prediction capability of our model.

## 2 Related Works

### 2.1 DL Applications in Survival Prediction

The canonical statistical ML algorithms usually use the clinical information we have mentioned above as covariates to make prediction. To get the most from high-throughput genomics data and medical image data, many deep-learning-based methods were proposed for survival prediction. We will review the literature about DL applications in survival prediction in the following, and the more refined branch of this, that is, using CNN with gene expression data, will be reviewed in the next subsection. Travers et al. proposed Cox-nnet (Ching et al., 2018), which is an ANN using high-throughput omics data as input; the hidden node features learned by neural network layers were seen as the dimension-reduced omics features, and a Cox regression layer was added to perform the final prognosis prediction. Compared with Cox regression, Cox-nnet could reveal more relevant biological information. Katzman et al. proposed DeepSurv (Katzman et al., 2018) to perform survival analysis; the architecture of DeepSurv consisted of some neural network layers and a linear output layer; the clinical data were used as input. What the DeepSurv predicted was the hazard ratio of a specific time, so that DeepSurv is a DL survival prediction model which is subjected to the Cox proportional hazard assumption. Results showed that DeepSurv outperformed the Cox regression model. Arya and Saha (2021) proposed a multimodal DL method for breast cancer survival prediction, and the data they used included genomics data, histopathology images, and clinical data. Their model was a gated attentive DL model with the random forest classifier stacked. Using this proposed method, they got a significant enhancement in sensitivity scores in the survival prediction of breast cancer patients. Panagiotis et al. proposed to mine the MGMT methylation status through MR images; they used a pretrained ResNet-50, which is a 50-layer residual network for transfer learning and outperformed the ResNet-18 and ResNet-34 (Korfiatis et al., 2017). Sairam et al. proposed to make pan-renal cell carcinoma classification and survival prediction from histopathology images using CNN and achieved good results in classification accuracy (Tabibu et al., 2019).

### 2.2 Using CNN With Gene Expression Data

CNN (Lawrence et al., 1997) is a kind of DL algorithm. In particular, CNNs using 2-D convolution kernels can be seen as a sort of tailor-made models for learning image representations; they can perform multiple computer vision tasks, such as image classification, face recognition, video recognition, image segmentation, and medical image processing. A canonical CNN usually has an input layer for loading the images. Behind the input layer, there are some hidden layers for image representation learning. At the end, an output layer will be added for making prediction. The hidden layers are mainly composed of (1) convolution layers which convolve the input, (2) pooling layers which reduce the dimensions of the data delivered by convolution layers, and (3) fully connected layers for learning the representations to be used for the final prediction. In the past decade, CNNs have made remarkable achievements, a cornucopia of great models based on CNN have been proposed, such as LeNet (LeCun et al., 1989), AlexNet (Krizhevsky et al., 2012), VGGNet (Simonyan and Zisserman, 2014), GoogleNet (Szegedy et al., 2015), and ResNet (He et al., 2016).

Training the CNN model with gene expression data may seem not workable subconsciously, because unlike the pixels in image data, which are in order, the gene expression data are much noisier and without order. To tackle this defect, some researchers committed to rearrange the gene expression data and use them for prediction based on CNN. Lyu et al. proposed the first model to convert gene expression data to image and make cancer-type classification with CNN (Lyu and Haque, 2018); they rearranged the normalized RNA-Seq counts into a matrix according to their relative position according to their chromosome numbers; their model achieved an accuracy score of up to 0.9559. Ma et al. proposed a model called OmicsMapNet; in this work, they transformed gene expression data into image by constructing a treemap graph using their functional annotation in KEGG BRITE dataset. And a CNN model was used to do prediction (Ma and Zhang, 2018). Guillermo et al. also proposed a method to rearrange gene expression data image by the treemap and KEGG BRITE dataset (López-García et al., 2020), but their method has a distinction from OmicsMapNet; that is, the area size of each functional branch in the treemap is determined by the gene expression levels in this branch, which makes the image more representative in terms of gene expression values. They used CNN to predict the 230 days of lung cancer progression-free survival (LUAD and LUSC), and transfer learning was added to increase the performance. Results showed that their method outperformed the ML algorithms and multilayer perceptron (MLP). Sharma et al. (2019) proposed Deep-Insight, a novel method in which the feature vector such as gene expression values is first fitted by clustering methods such as kPCA and tSNE and then the scatter diagram produced by clustering would be contracted to the smallest rectangle consisting of all the data points to get the final image. Their method performed well on the classification task using CNN. Bazgir et al. (2020) proposed a method to transform features to image based on their neighborhood dependencies, and CNN was used for drug resistance prediction. Oh et al. (2021) proposed PathCNN, which used multi-omics data and pathway data to predict 2-year OS for glioblastoma (GBM). They first convert the multi-omics data into images with 146 pathways. Then they leveraged CNN for 2-year OS prediction and got an average AUC of up to 75.5% for GBM.

## 3 Materials and Methods

**FIGURE 1:**
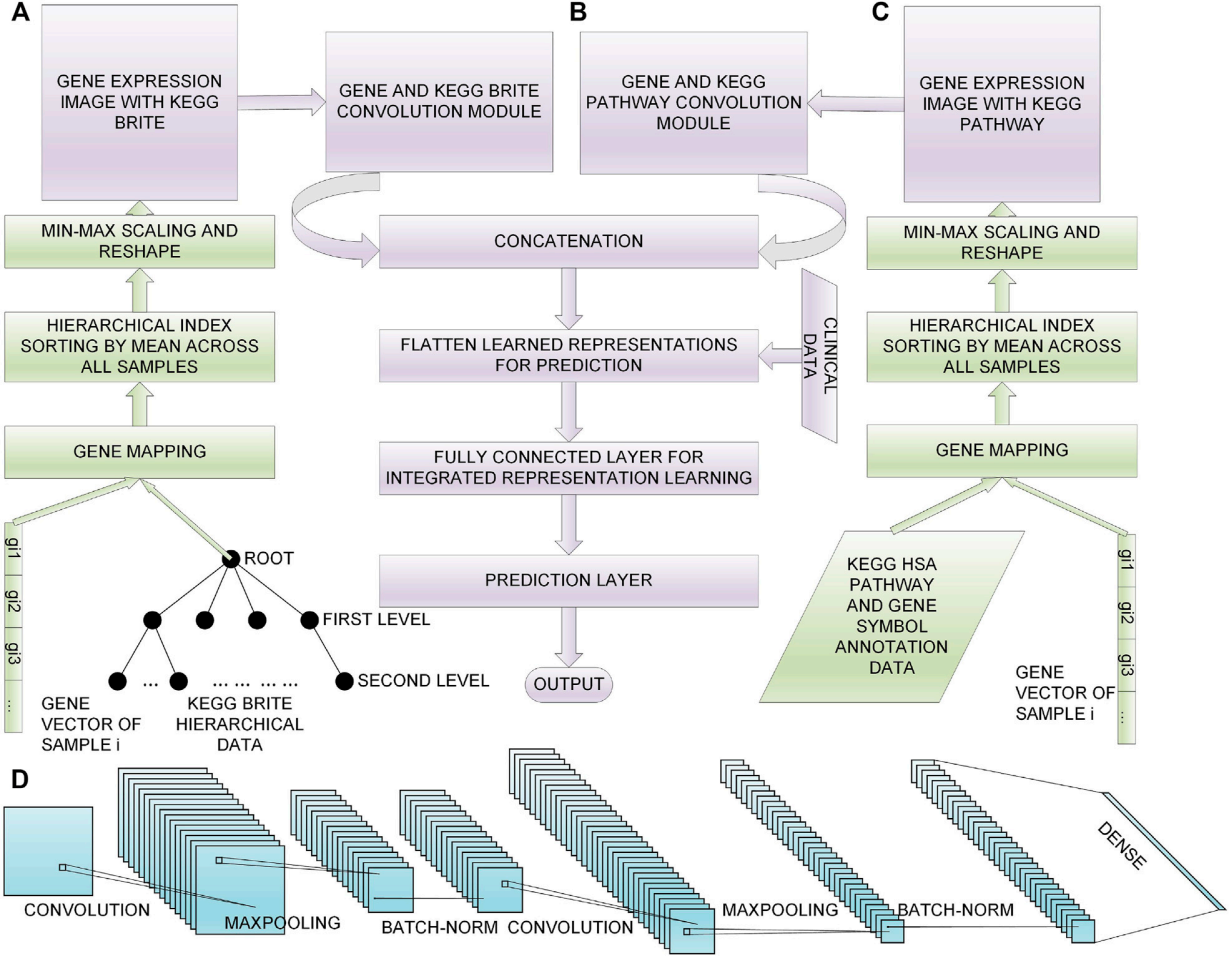
Overall process of the lung cancer long-term survival prediction: **(A)** the process of generating the gene expression image with KEGG BRITE data, **(B)** the DL model we propose for the prediction task, **(C)** the process of generating the gene expression image with KEGG Pathway data, **(D)** the detailed architecture of the convolution module we design for learning representations from the gene expression image.

In this section, we first give descriptions of the data sets we chose, then we introduce the process of feature selection; afterwards, we introduce our proposed method to convert the selected genes to gene expression images with KEGG BRITE and KEGG Pathway data incorporated, respectively (Figures 1A and 1C); finally, we present our multimodal DL model for 60 month lung cancer OS prediction (Figure 1B). The overview of the workflow is shown in Figure 1. The implementation of our method is available at https://github.com/PPDPQ/Lung-cancer-long-term-survival-prediction.

### 3.1 Data Descriptions

In this paper, we used the TCGA Pan-Cancer dataset (Chang et al., 2013; Tomczak et al., 2015) downloaded from the UCSC Xena data browser; from the data set, 1,122 lung cancer (LUAD and LUSC) samples were selected; then their gene expression data and clinical data were separated from the Pan-Cancer dataset, and 471 samples were selected for our research for they have all the data we need. To check the generalization performance of our model, we used a data set from the GEO database (Barrett et al., 2012) with accession number GSE37745, which have 196 samples. Of these, 195 samples were selected. In the 471 TCGA samples and the 195 GSE37745 samples we used in this paper, there are no duplicates between patients and samples. The KEGG BRITE gene function hierarchical data were download from http://rest.kegg.jp/get/br:br08902, and we chose the Genes and Proteins subsection for usage. There were three other datasets used for mapping genes to gene functions: (1) a table for mapping KEGG gene IDs to KEGG BRITE IDs was downloaded from http://rest.kegg.jp/link/hsa/brite; (2) a table for mapping KEGG gene IDs to HUGO gene names was downloaded from http://rest.kegg.jp/list/hsa; and (3) a table for mapping HUGO gene names to ENSEMBL gene IDs was downloaded from http://ftp.ebi.ac.uk/pub/databases/genenames/hgnc/tsv/hgnc_complete_set.txt. For KEGG Pathway data, we used the R package KEGGREST (Tenenbaum et al., 2019), org.Hs.eg.db (Carlson et al., 2019), and tidyverse (Wickham et al., 2019) to get the KEGG Pathway data and made mappings between pathways and genes. The general statistic for the data sets included are shown in Table 1.

**TABLE 1:** The general statistic for the datasets analyzed. Stages I to IV are the tumor stages defined by the AJCC staging system (Edge and Compton, 2010).

	TCGA lung cancer data set	GSE37745 data set
Number of samples included	471	195
Median age	68	65
Median age survived after 5 years	68	63
Median age dead after 5 years	68	66
Number of samples with stage I or stage IA	88	40
Number of samples with stage IB	129	89
Number of samples with stage II or stage IIA	48	6
Number of samples with stage IIB	84	29
Number of samples with stage III or stage IIIA	84	21
Number of samples with stage IIIB	17	6
Number of samples with stage IV	21	4
Percentage of over 5 year OS	26.1%	41.5%
Percentage of failed 5 year OS	73.9%	58.5%

### 3.2 Feature Selection

After separating the lung cancer data from the Pan-Cancer dataset, we performed feature selection on the lung cancer gene expression data based on mutual information (MI). There are 60,498 gene expression values (log _2_(*TPM* + 0.001)-transformed values) for each TCGA lung cancer sample (ENSEMBL (Zerbino et al., 2018) provides different IDs for one gene that maps to different chromosomes) and 20,356 genes’ expression values for each sample in GSE37745. First of all, we filtered the genes that appear in both the TCGA samples and the GSE37745 samples, and we got 18,975 genes. Then we performed feature selection on the TCGA samples. We first removed the genes with variance below the assigned threshold; in this research, we set this threshold as 10. And 3,053 genes were obtained for further selection. Then we split the data into a train set (80% of the samples) and a test set (20% of the samples), and we calculated the MI scores between genes and the labels on the train set; the labels we used were in keeping with our target problem, namely, whether the sample survived after 60 months. The MI between two variables *X* and *Y* can be calculated as follows:

$$I\left(X;Y\right)=\int_{X}\int_{Y}p\left(x,y\right)\log\frac{p\left(x,y\right)}{p\left(x\right)p\left(y\right)}dxdy$$ (1)

where *p*(*x*, *y*) is the joint probability density of variable *X* and *Y* and *p*(*x*) and *p*(*y*) are marginal densities. We can observe that *X* and *Y* are completely unrelated when *p*(*x*, *y*) is equal to *p*(*x*)*p*(*y*), and the MI score will be zero. The X here is the gene expression values, and Y is the targets which are 0 or 1, which indicates whether the sample survived after 60 months. Then we chose the top *K* genes according to their MI scores, we tested the prediction performance of different sizes of *K*s, and finally, we selected *K* = 1,000 for further data conversion. In fact, a size of 1,000 is roughly the same magnitude as the number of lung cancer samples, which means the model will not be prone to overfitting in terms of feature dimensionality.

### 3.3 Converting Gene Expression Data Into Images

With the 1,000 selected genes, we proposed a multi-index-sorting-based method to convert gene expression data into images, and the biological knowledge was incorporated.

#### 3.3.1 Gene Expression Image Using KEGG BRITE

The overview of the process to convert gene expression data into images using KEGG BRITE data is shown in Figure 1A. Firstly, we mapped the KEGG BRITE IDs to KEGG gene IDs, the KEGG gene IDs were mapped to HUGO gene names, and finally, the HUGO gene names were mapped to ENSEMBL gene IDs. After the above work was done, we successfully bridged the gaps between the gene expression data and the KEGG BRITE data, and we got the hierarchical data with genes and proteins as the root and gene expression values as leaves. We used these hierarchical data to do multi-index sorting; in each subclass in the leaf level, the genes were arranged according to their average expression level across all the lung cancer samples. The obtained rearranged genes were filled into a square matrix, and Min-Max was leveraged to transform gene expression values into a range from 0 to 1 for feeding into the convolution layer. The Min-Max process is defined by

$$X_{scaled}=\frac{X-X_{\min}}{X_{\min}X_{\max}}$$ (2)

where *X* denotes the expression values of a gene overall samples and *X*_min_ and *X*_max_ denote the minimum and maximum expression values of this gene, respectively.

#### 3.3.2 Gene Expression Image Using the KEGG Pathway

The overview of the process to convert gene expression data into images using the KEGG Pathway data is shown in Figure 1C. We implemented this process using R; first, we used KEGGREST for KEGG information; we got the human KEGG pathways and their Entrez gene IDs, and then we mapped the Entrez IDs to HUGO gene names and ENSEMBL gene IDs using the R package org.Hs.eg.db. With the generated data of mappings between genes and pathways, the same multi-index sorting, genes-to-image rearrangement, and Min-Max normalization as above were carried out.

### 3.4 Multimodal DL Model

To make good use of the generated gene expression images to predict lung cancer long-term survival, we proposed a multimodal DL model that makes good use of the multimodal data to achieve a good result.

#### 3.4.1 Model Construction

The model contained four input layers; among them, two were gene expression images, namely, the KEGG BRITE image and the KEGG Pathway image, and the other two inputs were clinical data: one is the age at initial pathological diagnosis and another is the AJCC pathological tumor stage. Because of the non-numeric characteristic of the AJCC pathological tumor stage, we encoded the stages by adding five per stage from *Stage I* to *Stage IV* to leverage the data.

With the two gene expression images being fed into the model, two convolution modules with similar structures were constructed to learn representations of the two images; the detailed structure of the convolution module of our model is shown in Figure 1D, where each convolution module contained two Conv blocks, i.e., (1) a convolution layer for learning representations from the input features sparsely, (2) a max-pooling layer for representation dimensionality reduction, and (3) a batch normalization layer for preventing overfitting. After the stacked two Conv blocks, a fully connected layer was added to integrate the learned representations of all the filters.

The generated representations of the two images were then concatenated and flattened, and the two clinical data were also concatenated in. Then a set of fully connected layers were added to learn the integrated representations of these four kinds of features. In the end, a sigmoid layer was used for the final prediction. Thus, our lung cancer long-term prediction task can be seen as a classification task in which the model used four kinds of input data to predict whether a sample survived after 60 months. The following are the introductions of the four inputs:

**Gene-expression-image-BRITE**: The gene expression image constructed from gene expression data and KEGG BRITE hierarchical gene function data.

**Gene-expression-image-Pathway**: The gene expression image constructed from gene expression data and KEGG Pathway data.

**Age-at-initial-pathological-diagnosis**: The sample’s age when the sample was diagnosed with lung cancer. This is one of the two kinds of clinical data.

**AJCC-pathological-tumor-stage**: A stage value given by the AJCC staging system (Edge and Compton, 2010) which describes the amount and spread of cancer in a patient’s body. This is another of the two kinds of clinical data. We encoded the stages by adding five per stage from *Stage I* to *Stage IV* to leverage the data, which means we encoded *Stage I* as 5 and *Stage IB* as 10, and other stages were encoded by that analogy.

#### 3.4.2 Model Hyperparameter Searching With Bayesian Optimization and Grid Search

In order to get the best model of the proposed model architecture, we leveraged Bayesian optimization to search the best hyperparameters. Bayesian optimization (Snoek et al., 2012) is a method that uses Bayes theorem to regularize the search for finding the minimum or maximum value of the objective function. This paper took advantage of Bayesian optimization to search for the best set of hyperparameters with the maximum AUC score. From the view of train, test, and validation sets, in this paper, we only used one train–test split for hyperparameter searching. Then we used another 50 different train–test splits for computing the generalized performance scores. To avoid data leakage, in each experiment of the 50 experiments, we created a model with only the hyperparameters; all the trained hyperparameters were initialized and trained on its own train set, which means, for each model, we set the hyperparameters only once using one train–test split, and then we used this set of hyperparameters for another 50 train–test splits. We used this strategy to display the generalization power of our model. All the DL-based models ensured their hyperparameters from 100 times Bayesian optimization searching trials, and all the ML models ensured the hyperparameters from Grid Search. The hyperparameters we searched are listed in Table 2. All the DL models in the paper are with the same depth and similar structure, the only difference being that they have different numbers of inputs. For the ML models, we leveraged Grid Search, which can take all the hyperparameter combinations in the searching space into consideration. The searching spaces and searching results for all the DL and ML models are provided as a table in the Supplementary Material.

**TABLE 2:** The hyperparameter searching space of the DL models for searching with Bayesian optimization.

Hyperparameters for searching	
Hyperparameter	Options for searching
Conv-BRITE-filters-1	32, 40, 48, 56, 64
Conv-BRITE-filters-2	80, 96, 112, 128
Dense-BRITE-units	128, 144, 192, 256
Dropout-rate-BRITE	0.1, 0.2, 0.3
Conv-pathway-filters-1	32, 40, 48, 56, 64
Conv-pathway-filters-2	80, 96, 112, 128
Dense-pathway-units	128, 144, 192, 256
Dropout-rate-pathway	0.1, 0.2, 0.3
Dense-1-units	64, 128, 144, 192, 256
Dropout-rate-1	0.3, 0.4, 0.5
Dense-2-units	32, 64, 128
Dropout-rate-2	0.3, 0.4, 0.5
Learning-rate	0.001, 0.002, 0.003

## 4 Experiments and Results

In this section, we present a number of experiments to show the performance of our multimodal DL model. Firstly, we tested the effectiveness of the two proposed methods, which convert gene expression data into gene expression images, on lung cancer long-term survival prediction. Secondly, we proved that inputting the two kinds of images into one DL model simultaneously can improve prediction performance. Thirdly, we tested the effectiveness of the two kinds of clinical data respectively. Finally, we compared our model with five ML models to show our model’s remarkable performance, and we conducted independent validation on the GSE37745 data set. The results are shown in Table 3.

**TABLE 3:** Results of the five average metrics scores from 50 different train–test-split experiments (mean ± SD) on the TCGA lung cancer data set. The accuracy, precision, recall, and f1-score were calculated with the optimal threshold selected using Youden’s *J* statistic.

Models	Average scores of 50 experiments on TCGA datasets				
	AUC	Accuracy	Precision	Recall	F1-score
DL-four-inputs	**71.48 ± 4%**	**69.85 ± 6%**	**69.17 ± 11%**	**87.93 ± 4%**	**76.66 ± 6%**
DL-three-inputs-age	65.68 ± 4%	64.42 ± 8%	62.34 ± 15%	86.39 ± 4%	71.00 ± 10%
DL-three-inputs-stage	70.69 ± 4%	68.95 ± 7%	68.29 ± 14%	87.54 ± 4%	75.64 ± 8%
DL-two-inputs	65.16 ± 4%	62.82 ± 9%	59.31 ± 17%	87.22 ± 5%	68.72 ± 11%
DL-one-input-BRITE	63.58 ± 4%	62.74 ± 9%	61.03 ± 17%	85.13 ± 4%	69.32 ± 11%
DL-one-input-pathway	64.69 ± 4%	63.31 ± 8%	60.97 ± 17%	86.32 ± 5%	69.62 ± 11%
KNN	53.63 ± 5%	57.22 ± 11%	52.51 ± 23%	85.47 ± 6%	61.54 ± 16%
SVM	54.77 ± 5%	56.11 ± 11%	52.69 ± 23%	84.17 ± 7%	60.58 ± 18%
Random-forest	57.41 ± 6%	57.33 ± 12%	53.40 ± 24%	85.09 ± 7%	61.68 ± 18%
Logistic-regression	50.81 ± 5%	55.41 ± 15%	53.91 ± 29%	82.50 ± 8%	58.67 ± 25%
MLP	55.06 ± 5%	54.61 ± 11%	49.14 ± 21%	83.91 ± 5%	58.75 ± 17%

The bold values are the highest among all the models.

### 4.1 Experiments Settings

In this subsection, we introduced the experiments implemented in this paper.

#### 4.1.1 Lung Cancer Long-Term Survival Prediction Experiments on the TCGA Lung Cancer Dataset

To prove the prediction power of the DL model, we used six different DL models which have similar structures. But their inputs are different. We used these six DL models to prove the effectiveness of the two kinds of gene expression images and the two kinds of clinical data. We used five ML models to prove that the DL models are better. The models used in this paper were introduced as follows:

**DL-Four-Inputs**: A DL model with two kinds of gene expression images and two kinds of clinical data as inputs; this model was used to show the best performance of our method;

**DL-Three-Inputs-Age**: A DL model with two kinds of gene expression images and the age at initial pathological diagnosis as inputs; this model was used to show the effectiveness of the clinical data age;

**DL-Three-Inputs-Stage**: A DL model with two kinds of gene expression images and the AJCC pathological tumor stage as inputs; this model was used to show the effectiveness of the clinical data tumor stage;

**DL-Two-Inputs**: A DL model with two kinds of gene expression images as inputs; this model aimed to indicate that using the two kinds of gene expression images as inputs simultaneously will make the DL model achieve better results;

**DL-One-Input-BRITE**: A DL model with only the KEGG BRITE gene expression image as input; this model was used to show that the KEGG BRITE gene expression image with the DL model was better than all the ML models so that it could validate the effectiveness of our DL algorithm and this gene expression image formation method;

**DL-One-Input-Pathway**: A DL model with only the KEGG Pathway gene expression image as input; this model was used to show that the KEGG Pathway gene expression image with the DL model was better than all the ML models so that it could validate the effectiveness of our DL algorithm and this gene expression image formation method;

**KNN**: An ML model using the K-nearest-neighbor algorithm (Laaksonen and Oja, 1996);

**SVM**: An ML model using the support vector machine algorithm (Noble, 2006);

**Random-Forest**: An ML model using the random forest algorithm (Biau and Scornet, 2016);

**Logistic-Regression**: An ML model using the logistic regression algorithm (Wright, 1995);

**MLP**: An ML model using the multilayer perceptron, which is a kind of a feedforward ANN (Pal and Mitra, 1992).

#### 4.1.2 Survival Analysis on the TCGA Lung Cancer Data Set

To more directly perceive the prediction performance of our best DL model without clinical data, namely, the two-input DL model, we conducted Kaplan–Meier survival analysis on the two-input model and the five ML models. Firstly, for all the models, we fixed the data shuffling random state to the same value (random seed was set as 126 in this paper) to ensure that all the models made prediction on the same test data set. Then we let the trained models make a prediction on the test set. Finally, we separated the samples in the test set into two groups for each model, which were the high-risk group with samples having predicted values that are larger than the optimal threshold selected with Youden’s J statistic and the low-risk group with samples having predicted values that are smaller than the optimal threshold. We compared the analysis results, leveraging the log-rank test (Bland and Altman, 2004); the analysis of the six models can be seen in Figure 6. We also implemented the Cox-PH analysis (Fox and Weisberg, 2002). To get rid of the influence of the other factors such as age, we only selected the DL model without any clinical input, namely, the two-input DL model so that the only remaining factor was the 1,000 genes we selected. Then we created a binary variable: if the sample was predicted dead, the variable’s value was 1; otherwise, the value was 0. Finally, we conducted a univariate Cox-PH analysis using this binary variable. The hazard ratio of each model was then calculated; we show them in Table 4.

**TABLE 4:** Hazard ratio of each model calculated from the univariate proportional hazard analysis model.

Models	HR (95% CI)	*p*-value
DL-Two-Inputs	**4.00**	<0.01
KNN	2.22	<0.20
SVM	**4.00**	<0.20
Random-Forest	2.31	<0.10
Logistic-Regression	3.60	<0.01
MLP	2.77	<0.07

The bold values are the highest among all the models.

#### 4.1.3 Generalization Performance Validation on the Independent Data Set

It is important to show the generalization ability of the model. So we conducted an independent test on the data from a different platform. We chose a data set from the GEO database with accession number GSE37745. And 195 samples were included in our test experiments. The gene expression data on the TCGA database are obtained by RNA-Seq, while the gene expression data on the GEO database are obtained through Chip-Seq (Park, 2009). The different sequencing technologies make the gene expression data on these two databases different. Hence, if our proposed method is successful on the GEO database, we can prove that our method is generalized. We implemented all the experiments in the same way we had done on the TCGA lung cancer data set. And the results can be seen in Table 5.

**TABLE 5:** Results of the five average metrics scores from 50 different train–test-split experiments (mean ± SD) on the GEO GSE37745 data set. The accuracy, precision, recall, and f1-score were calculated with the optimal threshold selected using Youden’s *J* statistic.

Models	Average scores of 50 experiments on GEO datasets				
	AUC	Accuracy	Precision	Recall	F1-score
DL-four-inputs	**72.51 ± 6%**	**73.85 ± 6%**	**77.39 ± 14%**	79.26 ± 7%	**77.18 ± 7%**
DL-three-inputs-age	70.77 ± 5%	71.03 ± 5%	68.96 ± 17%	81.26 ± 7%	72.60 ± 9%
DL-three-inputs-stage	72.36 ± 6%	72.46 ± 6%	71.04 ± 16%	81.32 ± 7%	74.39 ± 8%
DL-two-inputs	69.74 ± 6%	69.74 ± 6%	65.30 ± 17%	82.33 ± 9%	70.58 ± 10%
DL-one-input-BRITE	68.88 ± 5%	70.56 ± 5%	70.52 ± 14%	79.10 ± 8%	73.16 ± 7%
DL-one-input-pathway	67.37 ± 5%	68.05 ± 5%	62.70 ± 15%	80.91 ± 9%	68.89 ± 8%
KNN	55.76 ± 8%	63.85 ± 9%	56.35 ± 26%	**82.35 ± 13%**	60.84 ± 20%
SVM	54.32 ± 8%	61.33 ± 6%	63.13 ± 23%	72.04 ± 10%	63.28 ± 15%
Random-forest	55.59 ± 8%	60.72 ± 7%	52.78 ± 23%	77.37 ± 11%	58.21 ± 17%
Logistic-regression	54.08 ± 8%	58.51 ± 7%	49.83 ± 24%	75.82 ± 11%	55.07 ± 17%
MLP	54.69 ± 8%	59.03 ± 7%	49.04 ± 24%	75.89 ± 9%	55.56 ± 15%

The bold values are the highest among all the models.

### 4.2 Sample Selection and Split

For lung cancer long-term survival prediction, we chose the samples according to their OS time and OS event in their clinical data, where if a sample had an OS time longer than 60 months, we labeled the sample as 0, and if a sample had an OS time shorter than 60 months and the OS event was equal to 1, we labeled the sample as 1; we removed samples which did not come under any of the above circumstances. Then the samples which did not have the two kinds of clinical data were removed. The removed samples had no event occurring, but their OS time was less than 60 months. So we could not use these samples for training because we could not label them. Finally, we got 471 samples from the TCGA lung cancer data set and 195 samples from the GEO data set with accession number GSE37745. In the TCGA lung cancer data set, 26% of the samples survived after 60 months, and 74% did not. In the GEO GSE37745 data set, 42% of the samples survived after 60 months, and 58% did not. Then, we split the samples into 50 different train sets and their corresponding test sets in which 80% of the samples were chosen for training and 20% of the samples for testing. To get generalized results, we made 50 different train–test splits of the samples by changing the shuffling random rate, also known as random seed, of the data before applying the split. With the 50 different splits, every model was trained for 50 times, and 50 scores per metric were obtained, and the average scores were used as the generalized results.

### 4.3 Evaluation Metrics and Optimal Threshold Selection

#### 4.3.1 Evaluation Metrics

Since lung cancer long-term survival prediction can be viewed as a binary classification problem, we chose area under the ROC curve (AUC) to evaluate the classification performance of models. AUC represents the probability of a random predicted positive value located in the right of a random predicted negative value. And there are a series of classification thresholds being included compared with accuracy’s and f1-score’s only one classification threshold. So AUC can better display the classification performance of a binary classification model compared with accuracy and f1-score. Besides AUC, we also computed the accuracy, precision, recall, and f1-score of each model using a curated optimal threshold (the optimal threshold selection method will be introduced in the next subsection); their values are calculated as follows:

$$Accuracy=\frac{TP+TN}{TP+FP+TN+FN}$$ (3)

$$Precision=\frac{TP}{TP+FP}$$ (4)

$$Recall=\frac{TP}{TP+FN}$$ (5)

$$F1Score=\frac{2PrecisionRecall}{Precision+Recall}$$ (6)

where TP, FP, TN, and FN are illustrated in Table 6. The following are the explanations of the other four metrics:

**TABLE 6:** The interpretation of TP, FP, TN, and FN. TP is the number of correctly predicted dead samples, TN is the number of correctly predicted survived samples, FP is the number of wrongly predicted dead samples, and FN is the number of wrongly predicted survived samples.

	Prediction		
Ground Truth	—	P	N
	P	TP	FN
	N	FP	TN

**Accuracy**: Accuracy represents the number of correctly classified samples over the total samples. In this paper, it is the number of correctly predicted long-term survival samples and the correctly predicted dead samples over the total samples.

**Precision**: It represents the number of the correctly predicted dead samples (TP) over all the predicted dead samples (TP + FP).

**Recall**: It represents the number of the correctly predicted dead samples (TP) over all the real dead samples (TP + FN).

**F1-Score**: F1-score is a metric which takes into account both precision and recall.

#### 4.3.2 Optimal Threshold Selection Based on Youden’s *J* Statistic

Because of the imbalance of our data (74% positive vs. 26% negative for the TCGA cohort and 58% positive vs. 42% negative for the GSE37745 cohort), it is often difficult for the metrics scores calculated with the default threshold to represent the model’s classification performance. Hence, selecting the optimal threshold is a good way to get good results. And Youden’s *J* statistic (Ruopp et al., 2008) was used in our experiments to tune the classification threshold. Youden’s *J* statistic is calculated from sensitivity and specificity; the whole calculation process is shown as follows:

$$Sensitivity=\frac{TP}{TP+FN}=TruePositiveRate\left(TPR\right)$$ (7)

$$Specificity=\frac{TN}{FP+TN}=1-FalsePositiveRate\left(FPR\right)$$ (8)

J=Sensitivity+Specificity−1=TPR−FPR (9)

and the series of (TPR, FPR) tuples with their corresponding thresholds can be gained from the ROC curve. We choose the threshold with the largest value of Youden’s *J* statistic for further calculating the final classification metrics scores.

### 4.4 Results Analysis

In this subsection, we analyzed the results from 50 experiments per model. For a better learning effect on an imbalanced classification task, all the DL and ML models used SMOTE (Chawla et al., 2002) to oversample the minority samples except for the KNN model (an error occurred when using SMOTE on it, so we used random oversampling instead). Then we performed a Kaplan–Meier survival analysis (Goel et al., 2010) on our best DL model and the five ML models to make the classification performance of our model more intuitive.

#### 4.4.1 Model Validity Analysis

We firstly tested the validity of the two kinds of gene expression images. We used two CNN models each with same architecture as the four-input model to test the prediction performance of the two kinds of gene expression images. To evaluate the effectiveness of the gene expression images well, the five ML models used the same selected 1,000 gene expression values which we used for generating images as input. The average AUCs were 63.58% for the model with KEGG BRITE images and 64.69% for the model with KEGG Pathway images. Both the AUCs of the two kinds of images were far better than those of the five ML models, showing that it was meaningful to convert gene expression data into images.

Then we tested the performance when the two kinds of images were inputted in one model simultaneously, and we got an AUC of 65.15%, which was better than both of the model using only one gene expression image as input. This result enlightened us that we could add more inputs to improve the performance.

Next, we tested the effectiveness of adding clinical data into the DL model. We proposed two models with three inputs: one used two kinds of images and age at the initial pathological diagnosis as inputs, and the other used two kinds of images and the numerical AJCC pathological tumor stage as inputs. Their AUCs were 65.68% and 70.69%, respectively; both of them outperformed the model with only the two kinds of expression images as inputs, so that we could conclude that the two kinds of clinical data were both helpful in improving prediction performance.

Naturally, in the end, we harvested the best AUC (71.48%) when we fed all four kinds of data into one model, which was a remarkable result given that the samples were imbalanced. And the four-input model achieved the best scores in accuracy, precision, recall, and f1-score calculated from the threshold with the largest value of Youden’s *J* statistic, which was a fantastic accomplishment.

**FIGURE 2:**
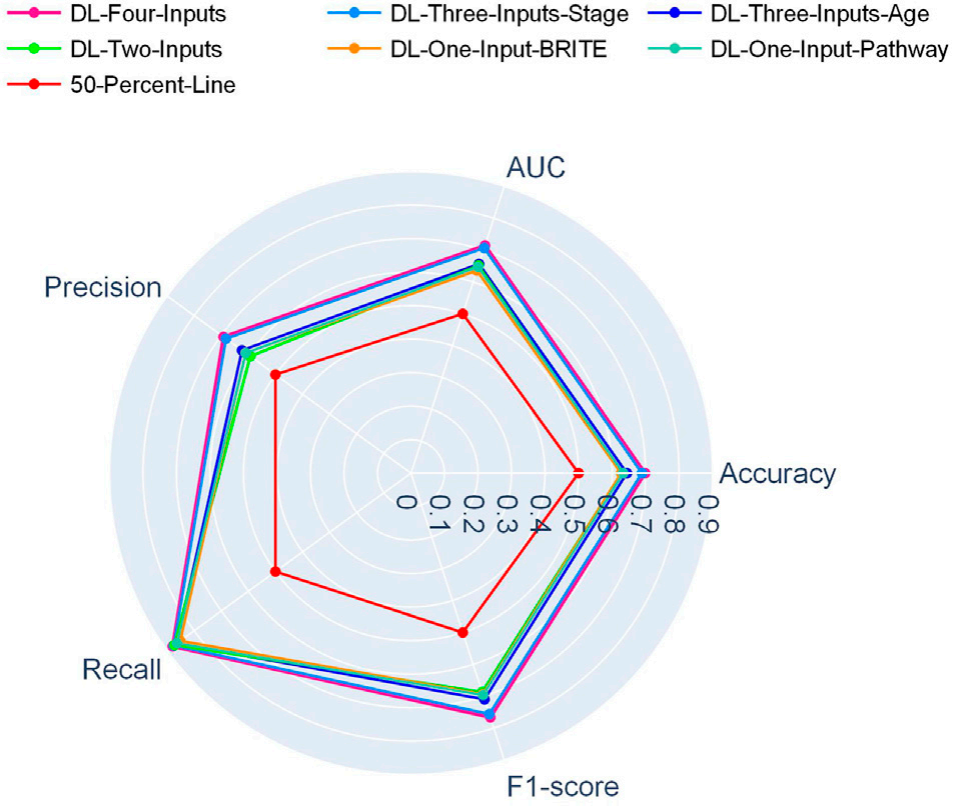
Radar plot for comparison of the DL models on the TCGA lung cancer data set.

In Figure 2, a radar plot showed the combination of the five evaluation metrics for the six DL-based models. It was readily observable that our best DL model, namely, the four-input model, achieved the best all-around performance among all the DL models.

**FIGURE 3:**
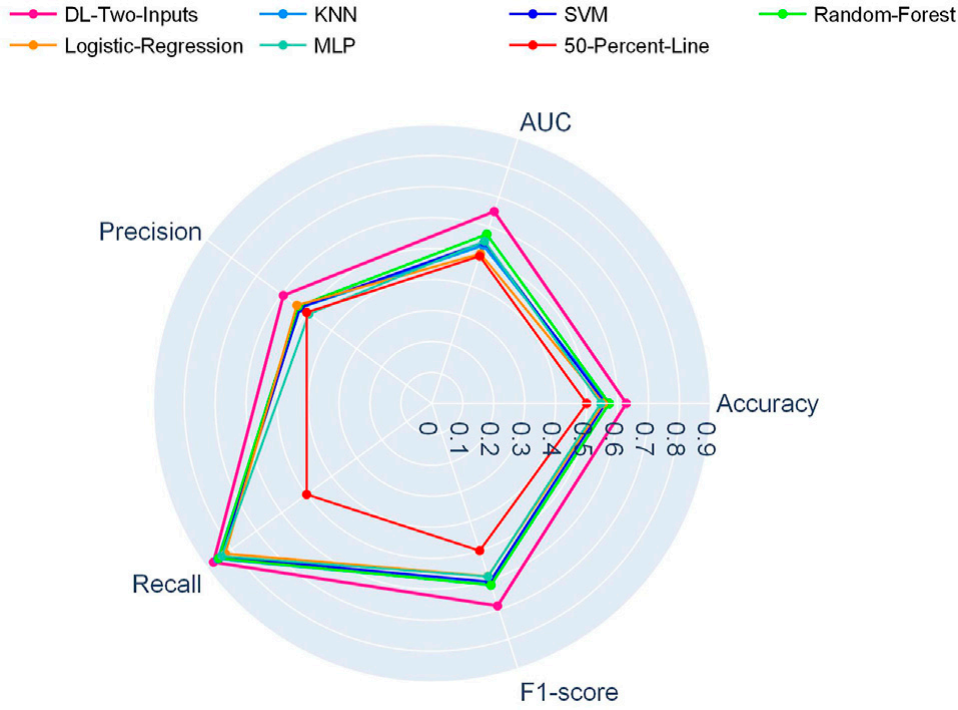
Radar plot for comparison of the two-input DL model with the ML models on the TCGA lung cancer data set.

And in Figure 3, another radar plot showed the synthetic performance of the five metrics for the two-input DL model and five ML models. We drew this radar plot aiming at making a performance comparison between the DL and ML models when no clinical data are included. And our two-input DL model performed better than all the ML models while not using any of the clinical data as input.

**FIGURE 4:**
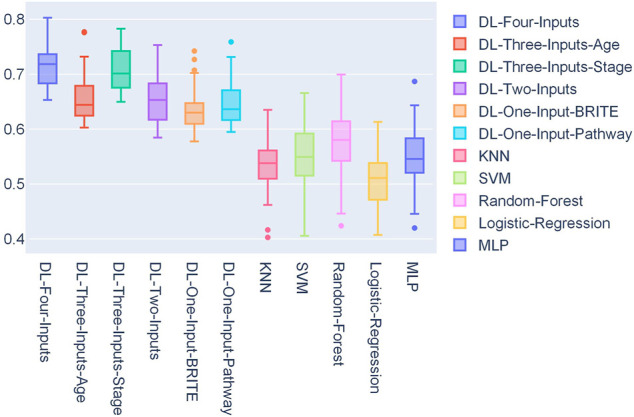
Box plot of the distribution of 50 AUCs for each model on the TCGA lung cancer data set.

In Figure 4, a box plot showed the distribution of AUCs from 50 experiments; we could observe that the four-input model was more robust for it got the best median value, first-quartile value, and third-quartile value among all the models. Among the ML models, we can observe that random forest performed the best.

**FIGURE 5:**
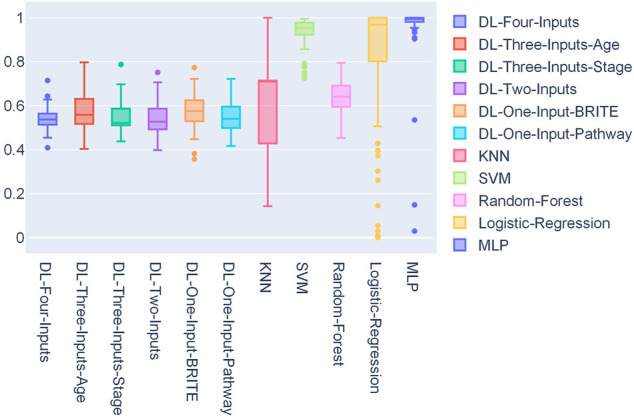
Box plot of the distribution of 50 thresholds for each model on the TCGA lung cancer data set.

We also conducted statistics on the 50 optimal thresholds for each model, and a box plot showing the distribution of the thresholds is presented in Figure 5. In this box plot, we can find that all the DL models have threshold distribution mainly between 0.4 and 0.6, so that the median values are closer to 0.5. With the fact that the TCGA lung cancer data set is very imbalanced, getting such threshold distributions indicated that the DL models overcame the problem of overfitting. As for ML models, we can find that their first-quartile values are closer to 1, which means that the ML models faced severe overfitting.

#### 4.4.2 Results of Survival Analysis on the TCGA Lung Cancer Data Set

**FIGURE 6:**
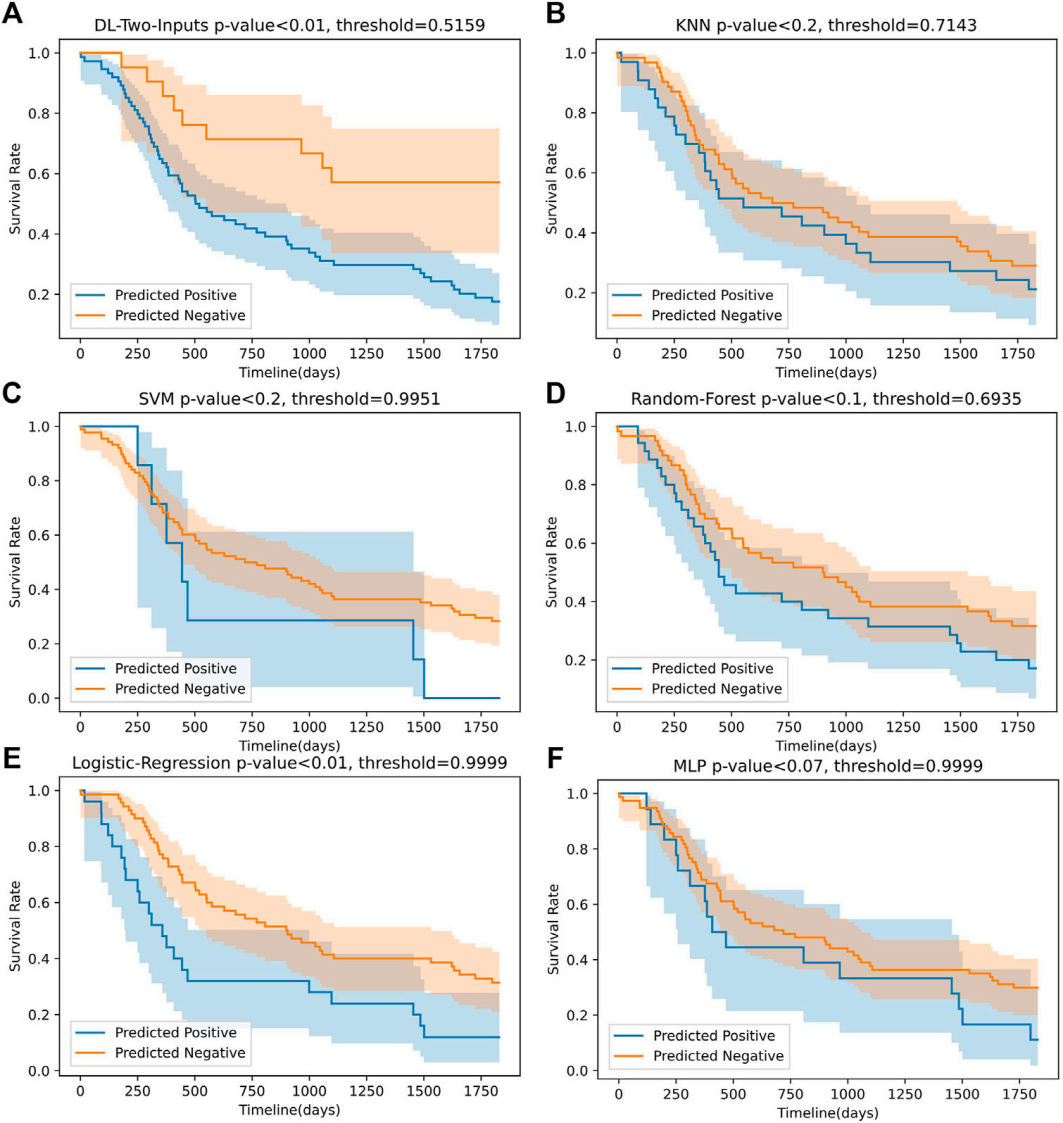
The Kaplan–Meier curves of the predicted high-risk and low-risk samples for our best DL model (without clinical data) and the five ML models on the TCGA lung cancer data set. The *p*-values were computed using log-rank test.

Figure 6 shows that the two-input model could divide the samples better than the other five ML models, and the two-input model got the smallest *p*-value among the models. As for the Cox-PH univariate analysis, in Table 4, we can observe that the DL model and SVM model both got a hazard ratio of 4.00, which means that the DL model and SVM model can separate the samples into two more distinct risk groups. But in Figure 6, we can see that the classification threshold of SVM was up to 0.9951 while the DL model’s threshold was 0.5159, which means that the DL model was far from overfitting, but the SVM was overfitting severely. All of these indicated that our DL model can better get two risk groups with more significant separation.

#### 4.4.3 Results of Generalization Performance Validation on the Independent Data Set

As can be seen with the results in Table 5, surprisingly, almost all the metric scores were higher than those of results on the TCGA lung cancer data set; even the total number of samples were much less than that of the TCGA samples. For example, the four-inputs DL model achieved 72.51% on AUC, larger than that of TCGA, which was 71.48%. The gap between DL models and ML models was more evident. We can see that the smallest AUC score was 67.37% of DL models, which was much larger than the best value of the ML models (55.76% with KNN). And the conclusion on the TCGA lung cancer data set is still effective on this independent data set. For instance, the four-inputs DL model was still the best among all the models, and the two-inputs DL model was still the best model without clinical data. All of above prove that our proposed method has the potential for generalization.

## 5 Discussion

In this paper, we introduced a method to predict lung cancer long-term OS using gene expression data and clinical data. Due to the extremely high feature dimensionality of gene expression data, it was difficult to directly use them in a DL or ML model for prediction. So we firstly used a supervised MI-based feature selection method to select the most relevant genes to the prediction target. Then we proposed a novel data transformation method to convert gene expression data into images with KEGG BRITE and KEGG Pathway data incorporated in. Using the gene expression images, we could take advantage of the CNN model to extract high-level representations from the gene expression data. The experiment results illustrated the effectiveness of using the CNN-based DL model with gene expression images to predict lung cancer long-term survival. When we combined two kinds of gene expression images as inputs into one DL model, we surprisingly found that the performance improved compared with the single-input DL model. This may be because with more input images, more biological knowledge was included, and the model got more trainable parameters while avoiding going deeper. To further improve the prediction performance, we added two kinds of clinical data into the model and achieved apparent performance improvement. Since the prediction task in this paper was essentially a binary classification problem, we chose AUC to better display the classification results. In order to make the results more intuitive, we also introduced accuracy, precision, recall, and f1-score into the paper. But we did a little special thing: we leveraged Youden’s *J* statistic to select the optimal classification threshold, so that we could get more accurate metric scores with the influence of imbalanced sample distribution being reduced. Besides the classification metrics scores, we conducted a Kaplan–Meier survival analysis to validate the effectiveness of our method, and the Kaplan–Meier curves of our model seemed more apparent for splitting the test set samples into two distinct risk groups, and the *p*-value calculated from the log-rank test was much smaller than the ML models. We did not intend to replace the methods in this field and just wanted to offer a novel solution to cope with high-dimensional gene expression data and to do cancer survival prediction.

Although our proposed method got remarkable results in average AUC, it still needed to be improved. We are planning to let the model be more interpretable; in the future, we will devote ourselves to finding the key genes or key pathways by tracing back to the weights of DL model layers or the gradients in back propagation. In conclusion, in this paper, we proposed a novel method to predict lung cancer long-term survival using a CNN-based DL model with well-designed gene expression images. Our method performed well, and it has great potential applications in cancer precision medicine.

## Data Availability

The original contributions presented in the study are included in the article/Supplementary Material, further inquiries can be directed to the corresponding authors.
