# Supplementary figures and images for: A Novel Deep Learning Method to Predict Lung Cancer Long-Term Survival With Biological Knowledge Incorporated Gene Expression Images and Clinical Data
Source: Front Genet. 2022 Mar 14;13:800853. doi: 10.3389/fgene.2022.800853 (PMC8964372; doi:10.3389/fgene.2022.800853)

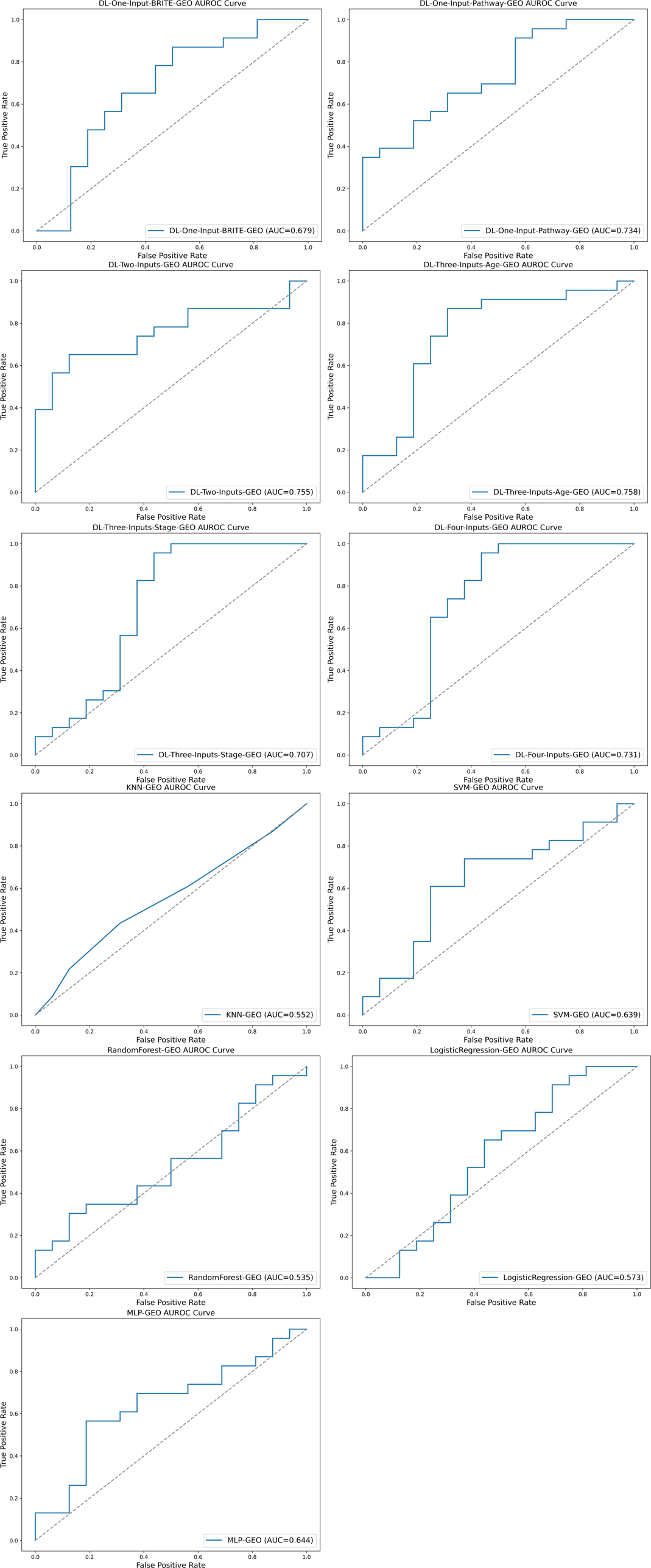

Supplement: Supplementary file 1 [file Image3.JPEG]

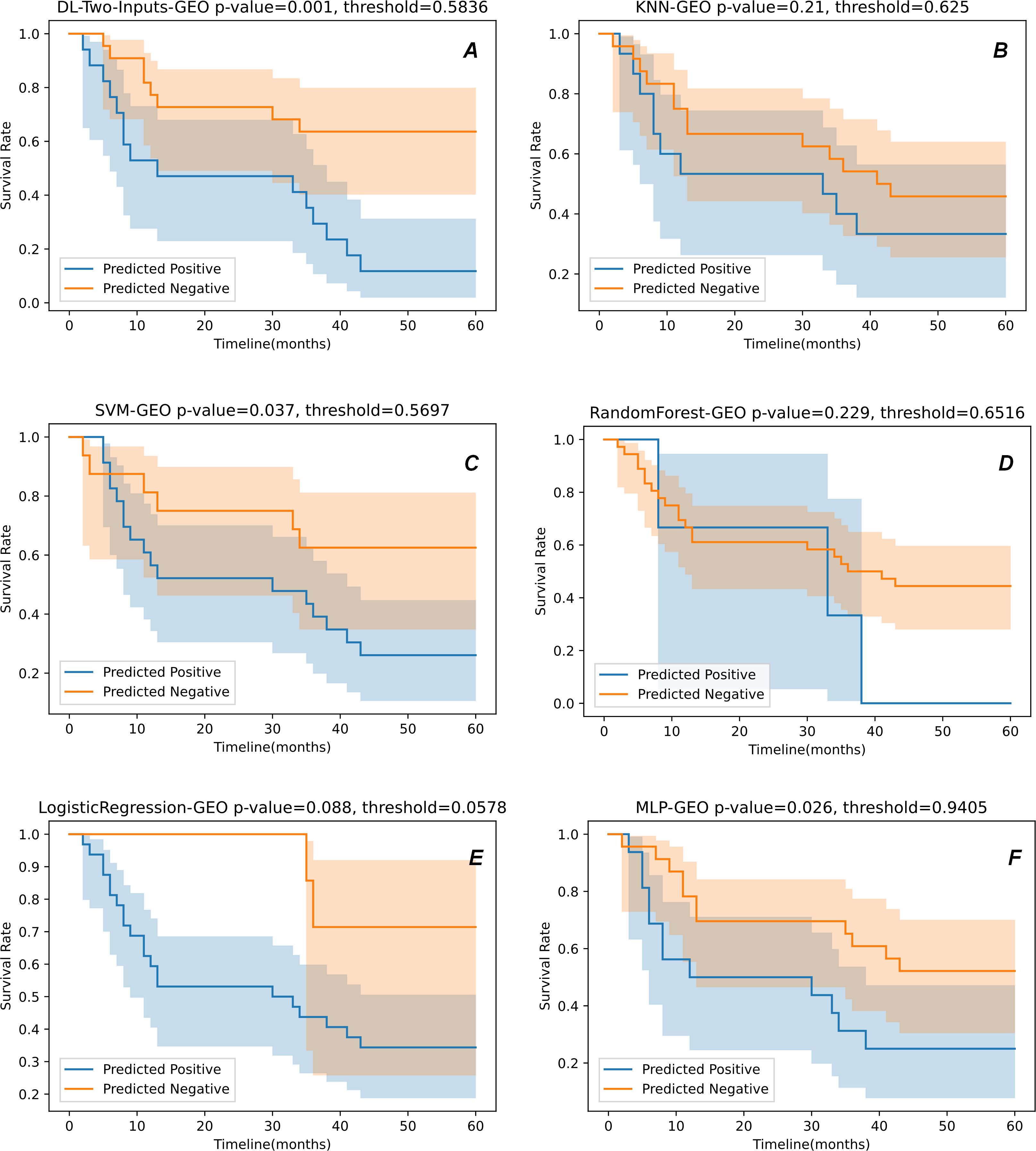

Supplement: Supplementary file 2 [file Image1.JPEG]

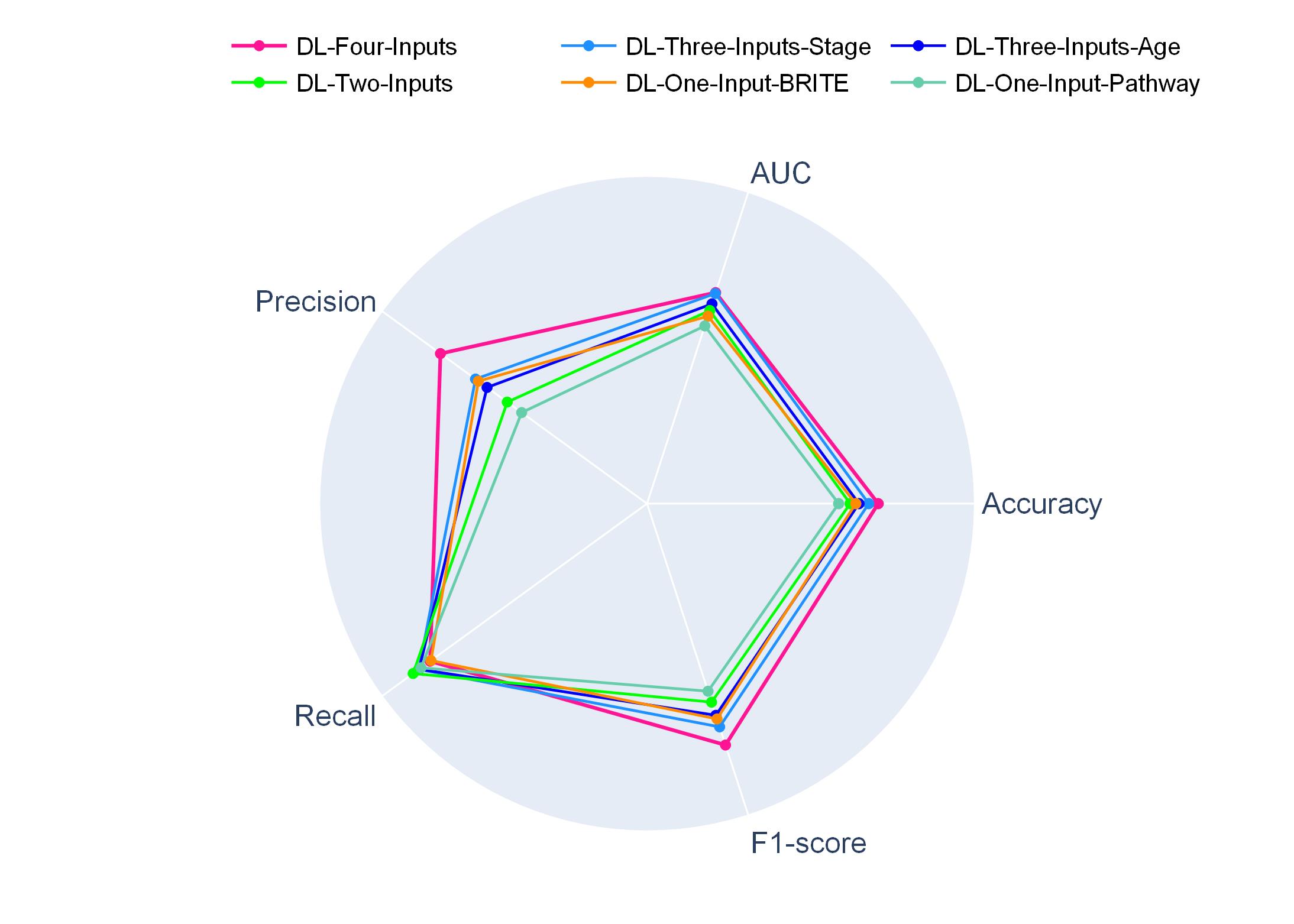

Supplement: Supplementary file 3 [file Image4.JPEG]

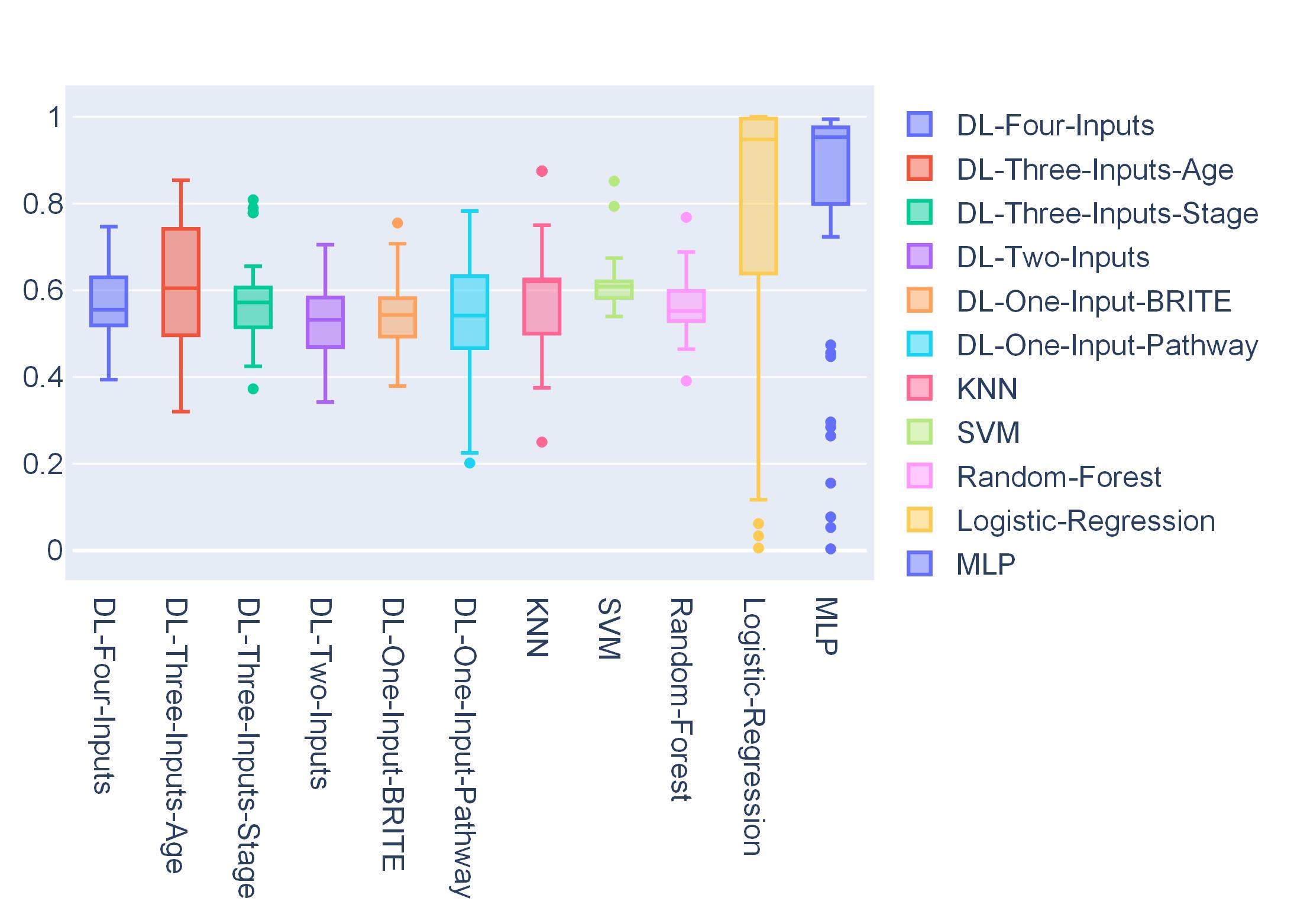

Supplement: Supplementary file 4 [file Image7.JPEG]

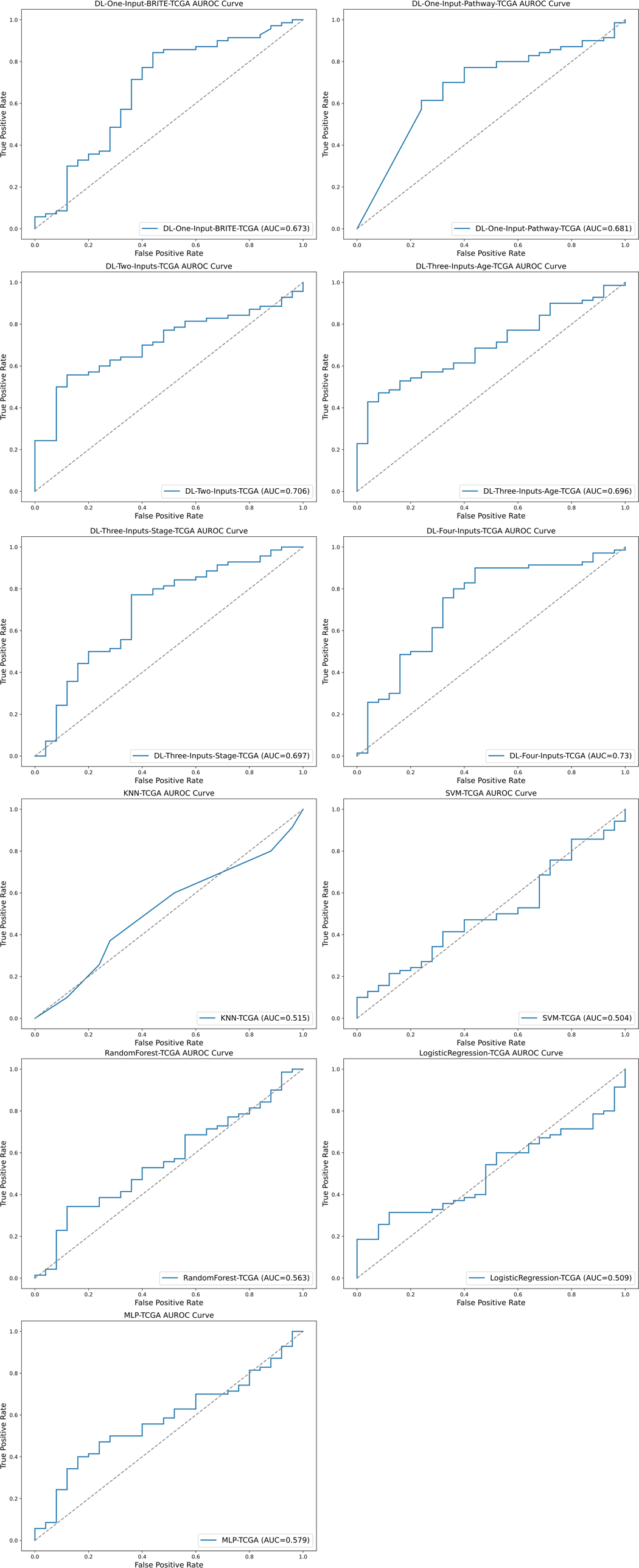

Supplement: Supplementary file 5 [file Image2.JPEG]

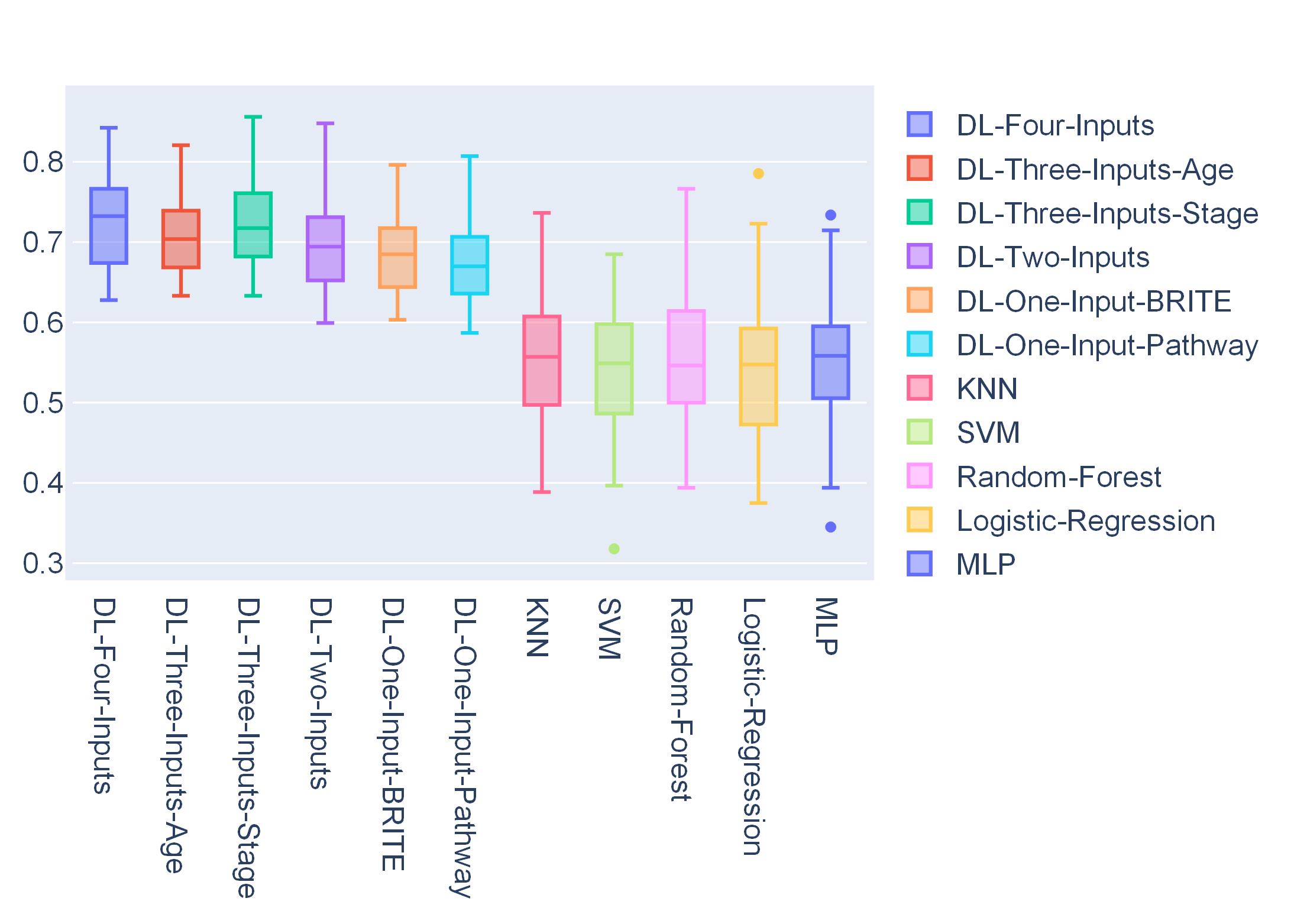

Supplement: Supplementary file 6 [file Image5.JPEG]

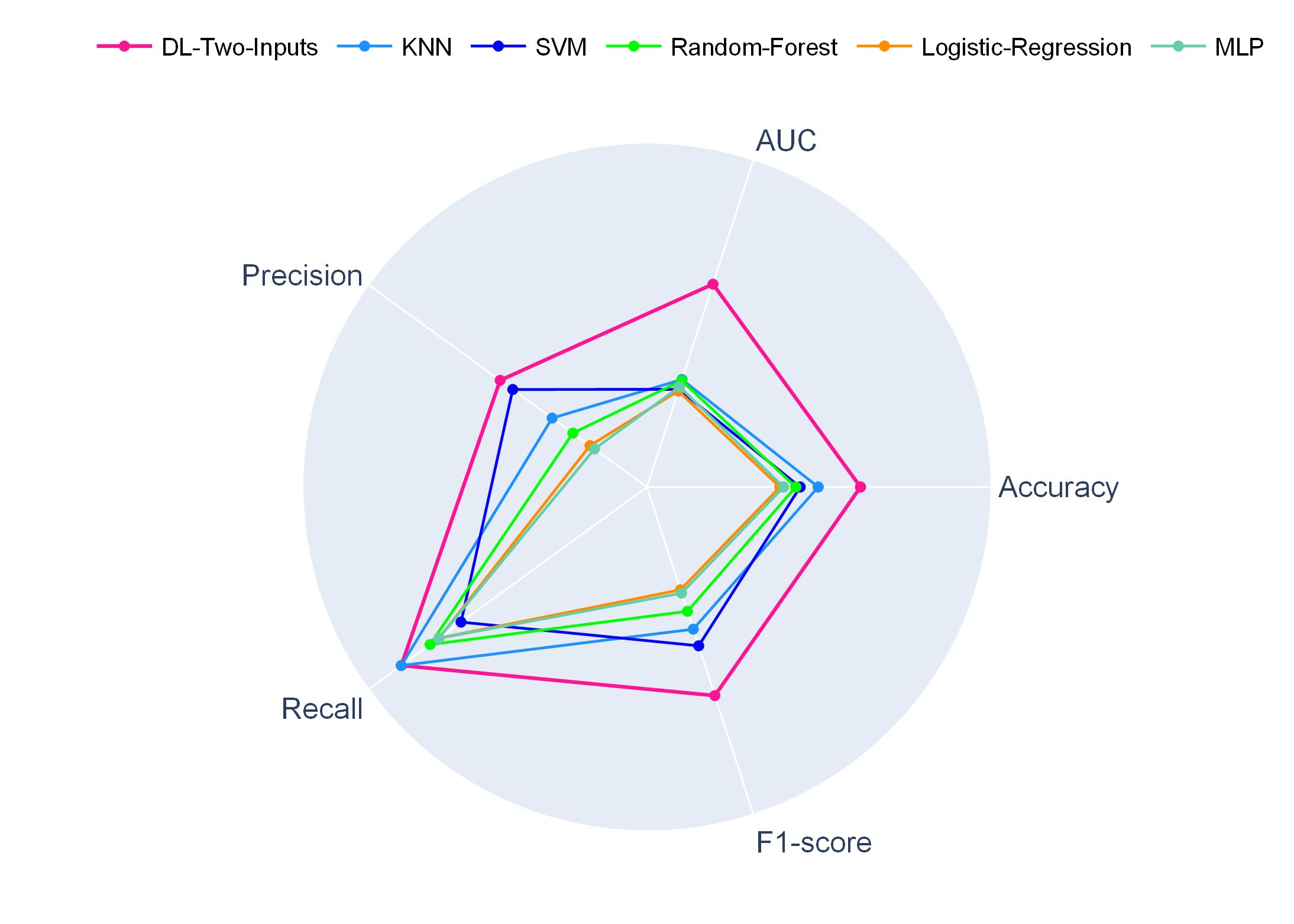

Supplement: Supplementary file 8 [file Image6.JPEG]
